# Characterization of Low-Density Polyethylene and LDPE-Based/Ethylene-Vinyl Acetate with Medium Content of Vinyl Acetate

**DOI:** 10.3390/polym13142352

**Published:** 2021-07-18

**Authors:** Nga Thi-Hong Pham

**Affiliations:** Faculty of Mechanical Engineering, HCMC University of Technology and Education, Ho Chi Minh City 71307, Vietnam; hongnga@hcmute.edu.vn; Tel.: +84-948691160

**Keywords:** EVA-28, low-density polyethylene, ethylene-vinyl acetate, elongation at break, tensile strength

## Abstract

Low-density polyethylene (LDPE) and ethylene vinyl acetate copolymer (EVA), which are non-polar and polar polymers, are immiscible and form a polyphase system. In this study, LDPE was mixed with 2.5%, 5%, 7.5%, 10%, 12.5% Ethylene-vinyl acetate (EVA-28) with a medium content of vinyl acetate (28% VA), respectively by injection molding machine and LDPE. Tensile strength and flexural strength were tested according to ASTM D638-02 standard and ISO 178 standard. The results showed that adding EVA-28 increased the elongation at break of the LDPE/2.5% EVA, LDPE/5% EVA and LDPE/10% EVA blend samples. In addition, the tensile and flexural strength of the LDPE/EVA blend decreases gradually as the EVA-28 content in the blend increases. The hardness decreases with the increasing EVA-28 content. EVA-28 spherical particles appeared scattered on the surface of the LDPE matrix, in the highest EVA-28 percent sample (12.5% EVA-28), the number of particles appeared to be quite a lot, and was dispersed quite evenly on the surface. The LDPE/EVA-28 blend achieved a higher elongation at the break than LDPE, in which 10% EVA-28 gives the highest elongation at break.

## 1. Introduction

The recycling and reuse of plastic waste films created from greenhouses is a global environmental problem. Agricultural greenhouse cover films and mulch films are typically made from low-density polyethylene (LDPE) [1,2,3]. Therefore, every year a large amount of LDPE film waste is found in agricultural waste streams. Recycling LDPE films from agriculture is a promising solution to reduce the amount of material discarded. However, since recycled LDPE exhibits moderate mechanical properties, which are further affected by an aging of the product, it is not really interesting for specific applications. New outlets for recycled LDPE could be developed if their low mechanical properties are improved by adding other materials. In fact, recycled LDPE has been widely used with virgin polymers to improve its mechanical properties. This is an effective way to reuse recycled LDPE. The addition of ethylene-vinyl acetate (EVA) to LDPE has been used commercially to increase resistance to environmental stress cracking, toughness, and resistance to film tearing [4,5,6].

LDPE is a thermoplastic, produced by a free radical polymerization method under pressure from 150–350 MPa and a temperature from 80–300 °C. The key characteristics of LDPE are low-temperature impact toughness, low-temperature impact resistance, good resistance to chemicals, and good creep resistance [7,8]. However, LDPE has some disadvantages such as low compatibility with additives, and high flammability. EVA is a combination of vinyl acetate (VA) and ethylene [9,10,11]. EVA has an important effect on blown plastic bags. Among the Polyethylene types, LDPE has many features that can be better compatible with EVA. Its branching structure and melting temperature are the same as EVA. For this reason, choosing LDPE blended with EVA can overtake the restrictions of each of the constituent polymers, contributing to increased mechanical properties [12,13,14]. The study of Bistra [15] on LDPE/EVA blends with 1.8%, 3.6%, 5.4%, 7.1%, and 8.9% EVA indicated that EVA were able to prevent the spread of cracking, hence adding EVA percentages will increase the toughness of the material due to increased adhesion strength. In Khonakdar’s work [16], rheological characteristics and morphology of LDPE/EVA blends with 0, 20%, 40%, 60%, 80%, and 100% EVA were investigated. Morphological examinations clearly reveal a two-phase morphology. Rheological parameters exhibit a linear trend, implying that the rheological parameters of the mixture obey the logarithmic mixing rule. However, when 20 wt.% EVA was added, no obvious change was observed that could be attributed to the phase separation of the system in this composition. Mechanical properties of binary LDPE and 0, 10%, 25%, 50%, 75%, 90%, 100% EVA blends were studied by M. Faker et al. [17] These results demonstrate that the viscosity and elasticity are higher in LDPE compared to EVA. This can lead to a better surface adhesion and efficient stress transfer from the substrate to the dispersed phase and thus 10%, and 25% EVA blends have a smoother morphology and a better distribution. Among them, the effect of co-crystallization was highest in the 10% EVA blend, which resulted in an increase in tensile strength and a decrease in elongation at break in this composition. Researcher Ray et al. [18,19,20] studied the stress–strain behavior of different blends of LDPE and EVA-28 with 0, 30, 50, 70, 100 wt.% EVA. It was found that tensile strength, yield stress and modulus tended to decrease with the increasing EVA content, while elongation et break exhibited slight fluctuations. Chung LEE et al. [21] studied 50% LDPE/50% EVA with 3.5, 9.5, 15, 19, 26 wt.% VA. The results showed that the elongation at the break of the LDPE/EVA blends showed the higher values, the more VA content contained in EVA, except for LDPE/EVA with 3.5% wt. VA. The values of elastic modulus were obviously decreased as the increasing VA content. This phenomenon is due to the mechanical characteristics of EVA. Research by Takidis [22] on the compatibility of LDPE and EVA-18 with 25%, 50%, and 75% EVA-18 showed that the tensile strength of LDPE/EVA blends increased almost linearly when increasing the EVA content, elongation at break of LDPE, EVA and blended materials LDPE/EVA were similar to tensile strength. Hoang has researched on the mechanical properties of EVA/LDPE films, showing that when there is 10% EVA in the EVA/LDPE blend, the elongation at the break of the film increases compared to films with only LDPE [23]. Thus, in the LDPE/EVA blend, the elongation at the break of the blend will rise linearly as the EVA ratio increases. Nevertheless, there are some studies showing that the adding EVA to the LDPE/EVA blend and the PE/EVA blend will turn down elongation at the break and impact on the mechanical properties.

## 2. Materials and Methods

### 2.1. Materials

LDPE resin (SABIC–LDPE 4024) with melt flow rate at 190 °C and 2.16 kg load is 4 g/10 min, density at 23 °C is 923 kg/m^3^ originated from Saudi Arabia, was supplied by Thuan Thang Plastic Co., Ltd, HCM City, Vietnam. EVA resin (EVATHENE^®^ UE639-04) with medium content of VA (28 wt.%), and specific gravity of 0.945 g/cm^3^ was supplied by Dong Nhan Phat Co., Ltd, HCM City, Vietnam. The formulation used for the blends are given in Table 1.

The blends of LDPE and EVA-25 resin were prepared using a TKC 2 vertical plastic injection molding machine at Dong Nhan Phat Co., Ltd., HCM City, Vietnam to make the samples. The mixed sample was poured into the machine for injection. The resin were pre-mixed using a plastic granule mixer before being poured into the injection molding hopper. Parameters of injection molding process: shaft temperature is 190–193 °C, die head temperature is 203–212 °C, cooling time is 8 s. The mold was kept at room temperature.

The test sample was free from oil, grease, and other impurities. Then it was left in the environment at the temperature of 23 ± 20 °C, humidity 50% ± 5%, for at least 40 h before carrying out the test.

### 2.2. Testing Methods

Determination the tensile strength for plastic materials according to the ASTM D638-02 standard. The number of tensile test samples included 5 samples/group of samples. The tensile test were performed using multimeter Shimadzu Autograph AG-X Plus 20 kN (Japan) machine at HCMC plastic Rubber Testing Center. High resolution camera was attached to the machine without contact with the sample and maximum test capacity of 20 kN, operating environment at 5–50 °C. The constant cross speed used was 50 mm/min at room temperature and mounted on the test machine.

Determination of the flexural strength according to the ISO 178 standard. The number of bend test samples were performed 5 times per group of samples. The Shore D hardness was determined according to the ASTM D2240-05 standard. The TECLOCK GS-702N hardness tester was used. To observe the microstructure, the device used was a HITACHI S-4800—high resolution scanning electron microscope (FeSEM), with acceleration 5.0 kV. The surfaces of the samples used for all SEM were sputtered with a platinum conductive layer before observation.

## 3. Results and Discussion

**Figure 1:**
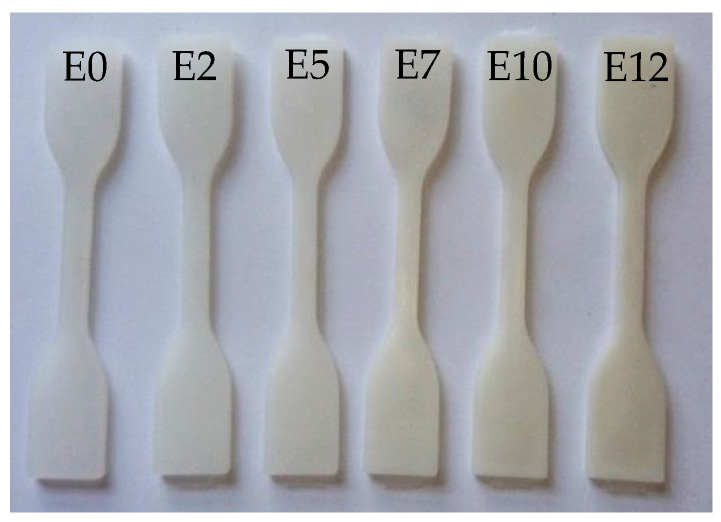
The samples after injecting.

Figure 1 presents the samples after injection. It can be seen that the less EVA-28 content is, the more transparent the specimen is. In which, sample E12 with the highest EVA-28 content (12.5%) is white and darker than the rest of the samples.

### 3.1. Tensile Strength

**Figure 2:**
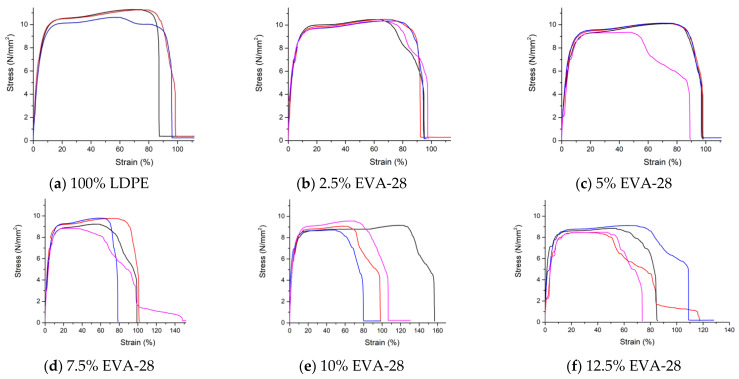
Stress–strain curves of tensile strength of LDPE/EVA samples.

Stress–strain curves of tensile strength of LDPE/EVA samples is shown as Figure 2. Table 2 shows the tensile strength of LDPE/EVA-28 mixture. From Table 2, it can be seen that the tensile strength of the blend decreases gradually as the EVA-28 percentage in the blend increases. The tensile strength of sample 100% LDPE is 9.71 MPa. When adding EVA, the tensile strength tends to decrease steadily. Specifically, the tensile strength is 9.08, 8.60, 8.09, 7.81, and 7.42 MPa, respectively, corresponding to EVA contents of 2.5%, 5%, 7.5%, 10%, 12.5% EVA-28. All these values are lower than neat LDPE. This result is similar to that of Ray [19]. When adding EVA to LDPE the tensile strength decreases. Specifically, the tensile strength of neat LDPE which is 15 MPa, reduced to 13 MPa when adding 30% EVA, and is further reduced to 11.2 MPa when adding 50% EVA. The composition has a marked effect on the tensile strength, and elongation at break. The chain slippage and orientation of chains along the drawing direction take place simultaneously. M. Faker [17] concluded that the mixing of LDPE and EVA reduces the melting point of the LDPE phase and increases the melting point of the EVA phase. The dilution effect of the EVA chain on the LDPE phase and the nucleation effect of the LDPE chain and LDPE crystal on the EVA phase are the reasons for the decrease in tensile strength.

Table 3 shows the average elongation at break of the samples (%). It can be seen that the elongation at break of the 100% LDPE sample is 92.70%. When EVA-28 is added to the LDPE/EVA blend, the elongation at break of the sample E2, E5 and E10 increases. In the LDPE/2.5% EVA-28 sample, the deformation increased to 93.84%, adding 5% EVA-28 continued to increase slightly (94.66%), then decreased to 92.25% for sample LDPE/7.5% EVA-28 blend. When the EVA-28 percentage continued to increase up to 10%, the elongation at break rose sharply to 107.97%, then decreased to 86.28% in sample LDPE/12.5% EVA-28 blend. Thus, when adding EVA-28 to the LDPE, the elongation at break of the blends increases compared to that of without EVA-28, except for sample E7 and sample E12. The oscillation trend of elongation at break is similar to that of Ray’s research [19]. When adding 30% EVA to LDPE, elongation at the break decreases compared to neat LDPE; however, elongation at the break increases when adding 50% EVA. Elongation at the break of LDPE/50% EVA is also higher than neat LDPE in the work of Lee [21]. According to M. Faker [17], the results of rheological studies show that the elasticity tends to increase while the tensile strength tends to decrease. This is explained by the higher efficiency of co-crystallization in that mixture. In addition, these variations of strength properties are very much dependent on the strain rate. The trend of variation of different properties observed at higher strain rates like 100 cm/min or 50 cm/min change significantly when the strain rate is reduced to 20 cm/min or 10 cm/min. 

The MFI provides important information on the flow characteristics of polymers. The flow indices (at 235 °C, 2.16 kg) of neat LDPE and its blend with 10% EVA are shown in Table 4. It can be seen that the addition of EVA in the LDPE matrix resulted in a decrease in the MFI. The MFI value of LDPE was 4.66 g/10 min and it was reduced to 3.98 g/10 min by adding 10 wt.% EVA. The decrease in MFI is attributed to the interaction between EVA and the polymer matrix and thereby reduces the mobility of the melted polymer.

### 3.2. Flexural Strength

**Figure 3:**
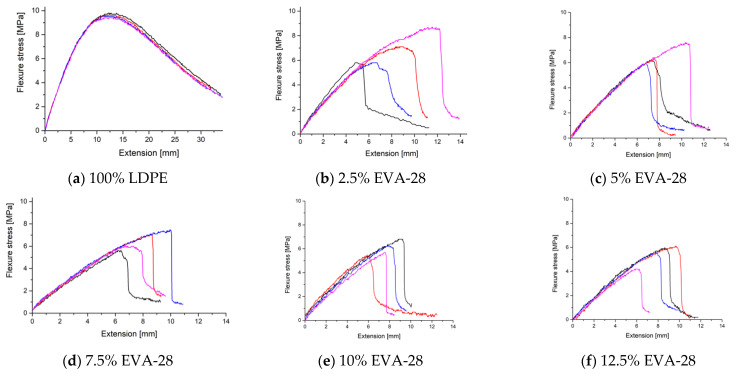
The flexural stress-strain curves of the LDPE/EVA mixtures.

Figure 3 presents the stress–strain curves of the flexural test of the LDPE/EVA blends. From the figure it can be seen that when adding EVA-28, the flexural strength decreases and elongation at break increases compared to other EVA-28 ratios in the blend. The reason for this is because it is similar to the tensile strength and because of the appearance of air bubbles during the pressing process, leading to unstable results.

Table 5 summarizes the results of measuring the flexural strength of the samples E0, E2, E5, E7, E10, and E12. Flexural strength decreased steadily from the sample E0 (8.46 MPa) to the sample E2 (5.71 MPa), continued to decrease to the sample E5 (5.34 MPa) and slightly increased in the sample E7 (5.39 MPa), then further decreased in the sample E10 (4.91 MPa) and the sample E12 (4.29 MPa). This shows that as the EVA-28 content in the blend increases, the flexural strength decreases. The decrease in flexural strength can be attributed to reduced stiffness, which is a result of uneven dispersion and distribution of EVA-28 particles in the polymer matrix and consequently interfering in the chains in the bonding. It was found that although the interfacial interaction in the melt state has an influential effect on the mechanical properties of the blends, the mechanical behavior of the semicrystalline polymer blends such as PE/EVA blends can also be affected by the crystallization process [17].

### 3.3. Hardness

**Figure 4:**
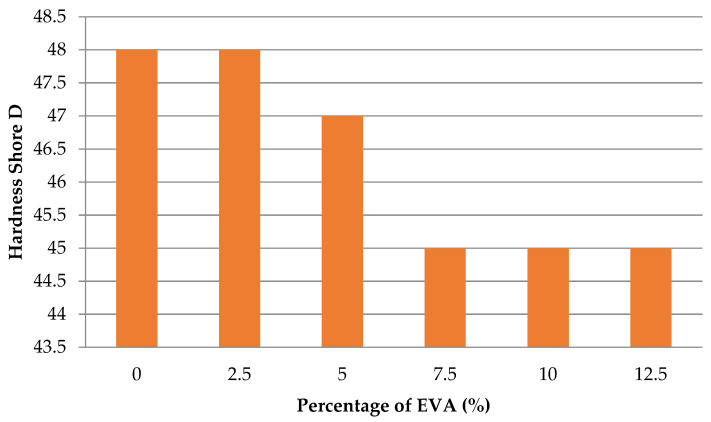
Hardness average values of test samples.

Figure 4 shows the average hardness values for each sample group. It can be seen that hardness decreases with increasing EVA-28 content. In the sample E0, the LDPE content was at 100%, the hardness of the sample was 48 Shore D. In the sample E2, the hardness did not change, then the hardness decreased slightly in the sample E5 with 47 Shore D, then decreased to 45 Shore D for the sample E7, E10, and E12.

### 3.4. SEM Micrographs

**Figure 5:**
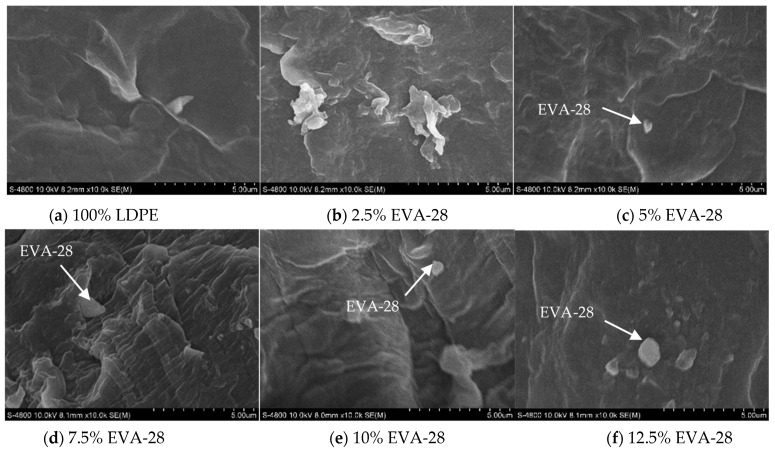
SEM microstructure of the LDPE/EVA mixtures.

To be able to better elaborate the property behavior of the LDPE/EVA mixture, this research analyzes the surface microstructure image of the blend. The results are shown in Figure 5. Looking at the SEM image results of the samples, it can be seen that the sample E0 is typical of the microstructure of LDPE. In the sample E2, there appeared EVA-28 spherical particles scattered on the surface of the LDPE matrix, in the sample E12 with the highest EVA-28 ratio of 12.5%, the number of particles appeared to be quite a lot. The number of EVA-28 particles increased from the sample E2 and to the sample E12 was dispersed quite evenly on the surface. The biphasic boundary is clear and the adhesion is relatively poor. For EVA, the presence of carbonyl groups in EVA increases its adhesion to the surfaces of various materials. EVA can mix well with additives and fillers at a high rate [6]. LDPE is a low-density polymer and, in the process of making samples, the mixing process is the interleaving of molecules of each polymer under mechanical action. The elongation at the break of EVA is higher than at LDPE, when the compatibility of LDPE and EVA in the blend of LDPE/EVA improves elongation at the break compared to samples containing 100% LDPE [9,20]. The enhancement of the LDPE and EVA interoperability was explained by chemical reactions occurring in the molten mixing process [10]. In Khonakdar’s study, it can be observed that LDPE/EVA blends exhibit a two-phase morphology, implying the immiscibility of the blends. The size of dispersed domains at 20 wt.% EVA content falls within the range of a submicron to about 2 µm [16]. SEM micrographs clearly indicate that all the blends with different compositions have a two-phase morphology, showing the immiscibility of the used PE and EVA, in whole composition ranges [17]. SEM micrograph of PE/EVA 90/10 blend shows the uniform distribution of submicron EVA particles in the PE matrix. Increasing the EVA content from 10% to 25% increases the average diameter of the dispersed EVA domains from 0.22 to 0.52 µm. It was also found that although the interfacial interaction in the melt state had an influential effect on the mechanical properties of the blends, the mechanical behavior of the semicrystalline polymer blends such as PE/EVA blends could also be affected by the crystallization process.

## 4. Conclusions

From the above test results, when adding EVA-28 to the LDPE/EVA mixture, the elongation at the break of the sample LDPE/2.5% EVA-28, LDPE/5% EVA-28 and LDPE/10% EVA-28 increases. This demonstrates EVA’s potential application in the processing of plastic packaging products, agricultural greenhouse covering and mulching films. The tensile strength of the LDPE/EVA mixture decreases gradually when increasing the EVA-28 content in the blend.

Flexural strength of the LDPE/EVA mixture decreases linearly when extending the EVA-28 content in the blend. The hardness decreases with the increasing EVA-28 content: the hardness of 100% LDPE and LDPE/2.5% EVA-28 were 48 Shore D, and then the hardness decreased slightly to 47 Shore D in LDPE/5% EVA-28 and fell to 45 Shore D for the rest of the sample.

EVA spherical particles appeared scattered on the surface of the LDPE matrix, in the highest EVA-28 percent sample (12.5% EVA-28), the number of particles appeared to be quite a lot, and was dispersed quite evenly on the surface. The biphasic boundary is clear and the adhesion is relatively poor.

## Figures and Tables

**Table 1:** Formulation used for the LDPE/ EVA-28 blends.

Sample	E0	E2	E5	E7	E10	E12
LDPE (wt.%)	100	97.5	95	92.5	90	87.5
EVA-28 (EVA with 28 wt.% VA)	0	2.5	5	7.5	10	12.5

**Table 2:** Average results of tensile strength (MPa).

Sample	E0	E2	E5	E7	E10	E12
Tensile strength (MPa)	9.95	9.14	8.76	7.91	7.84	7.54
	-	9.16	8.81	8.44	7.77	7.15
	9.91	9.02	8.81	8.47	7.40	7.80
	9.28	9.02	8.03	7.53	8.24	7.17
Average (MPa)	9.71	9.08	8.60	8.09	7.81	7.42
Standard Deviation	0.38	0.08	0.38	0.45	0.34	0.31

**Table 3:** Average elongation at break of the samples (%).

Sample	E0	E2	E5	E7	E10	E12
Elongation at break (%)	85.68	93.91	96.65	96.08	153.44	82.74
	-	91.23	97.32	99.18	95.24	82.62
	96.73	93.65	96.19	77.7	77.45	107
	95.69	96.59	88.5	96.04	105.73	72.75
Average (%)	92.70	93.84	94.66	92.25	107.97	86.28
Standard Deviation	6.10	2.19	4.14	9.81	32.49	14.58

**Table 4:** Results of Melt Flow Index.

Sample	MFI (g/10 min)										Average (g/10 min)
E0	4.69	4.61	4.61	4.68	4.62	4.68	4.64	4.67	4.66	4.68	4.66
E10	4.01	4.21	4.07	3.52	4.04	4.01	4.06	4.04	4.03	3.99	3.98

**Table 5:** Average results of flexural strength (MPa).

Sample	E0	E2	E5	E7	E10	E12
Flexural strength (MPa)	9.95	9.14	8.76	7.91	7.84	7.54
	9.65	9.16	8.81	8.44	7.77	7.15
	9.91	9.02	8.81	8.47	7.40	7.80
	9.28	9.02	8.03	7.53	8.24	7.17
Average (MPa)	9.70	9.08	8.60	8.09	7.81	7.42
Standard Deviation	0.31	0.71	1.32	1.92	2.2	2.7

## Data Availability

The data used to support the findings of this study are available from the corresponding author upon request.
